# Supplementary material for: The common origin of symmetry and structure in genetic sequences
Source: Sci Rep. 2018 Oct 25;8:15817. doi: 10.1038/s41598-018-34136-w (PMC6202410; doi:10.1038/s41598-018-34136-w)
Supplement: Supplementary file 1 — Supplementary Information [file 41598_2018_34136_MOESM1_ESM.pdf]

# Supplementary Information

*The common origin of symmetry and structure in genetic sequences*

*Giampaolo Cristadoro, Mirko Degli Esposti, Eduardo G. Altmann*

# 1 Derivation of the nested hierarchy of symmetries

We derive the nested hierarchy of symmetries in the minimal model for genetic sequence.

## 1.1 Notation and model properties

To fix notations, we describe our model and its statistical properties as follows:

- The full sequence is build concatenating $r$ macro-structures: $\mathbf{s} = \mathbf{m}_1\, \mathbf{m}_2 \cdots \mathbf{m}_r$.
- A macrostructure $\mathbf{m}$ is build concatenating $m$ domains: $\mathbf{m} = \mathbf{d}_1^{\mathbf{m}}\, \mathbf{d}_2^{\mathbf{m}} \cdots \mathbf{d}_m^{\mathbf{m}}$.
- The average domain length is denoted by $L_D$, the average macro-structure length is denoted by $L_M$. The total length of the sequence is $N$.
- A domain $\mathbf{d}^{\mathbf{m}}$ in the macro-structure $\mathbf{m}$ is a finite-size realisation of a process chosen between two[1] symmetrically related process-types: $C_{\mathbf{m}}$ and $\hat{C}_{\mathbf{m}}$. We use the notation $\mathbf{d} \in C$ to indicate that $\mathbf{d}$ is generated by the process of type $C$.

[1] Generalisations to more than two symmetrically domain-types is straightforward and it is not expected to change the main features of the model.

- For a a given observable X, we denote by $f_C(X)$ the limiting relative frequency[2] of occurrence of $X$ in a domain of type $C$. Recall that, the definition of symmetrically related processes (of the same macro-structure) imposes that, for every choice of $X$:

$$f_C(X) = f_{\hat{C}}(\hat{X}) \tag{1}$$

  In principle, different macro-structures have different process-types statistics.

- We denote by $(\underline{c}, \underline{l})$ an ordered sequence of domains of types $\underline{c} := (c_1, \cdots, c_k)$; $\quad c_j \in \{C, \hat{C}\}$ of lengths $\underline{l} := (l_1, \cdots, l_k)$ respectively; and by $\widehat{(\underline{c}, \underline{l})}$ the sequence of domains defined by $(\hat{c}_k, \cdots, \hat{c}_1)$ and $(l_k, \cdots, l_1)$. We denote by $\pi^{\mathbf{m}}[(\underline{c}, \underline{l})]$ the relative frequency[3] of counts of subsequence of domains $(\underline{c}, \underline{l})$ in **m**.

  We denote by $\pi^{\mathbf{m}}(\{c, l\})$ the relative frequency of a cluster of length $l$ of domains of the same type $c$.
  For $\alpha, \beta \in \{C, \hat{C}\}$ and $k \geq 1$ , we denote $\pi^{\mathbf{m}}(\alpha, \beta; k)$ the relative frequency of $j$ such that $\mathbf{d}_j \in \alpha$ and $\mathbf{d}_{j+k} \in \beta$.

- In each macro-structure, the probability distribution of domain-sizes is denoted $p_{\mathbf{m}}(l)$.

- We do not enforce any prescription to concatenate domains in a macrostructure (determined by $\pi$), but the following properties:

  - $\pi^{\mathbf{m}}[(\underline{c}, \underline{l})] = \pi^{\mathbf{m}}[\widehat{(\underline{c}, \underline{l})}]$ This ensure that the structural statistics of two symmetrically coupled domain-types ordering is unbiased.

[2] We assume that process types $C$ are such that $f_C(X)$ are well defined for all choice of observables $X$ in the limit of size of domains going to infinity.

[3] We assume that the structural properties of a given macro-structure is such that $\pi$ is well defined in the limit of number of domains $m$ going to infinity.

– for $k >> L_S/L_D$ ; $\pi^{\mathsf{m}}(c_1, c_2, k) = \pi^{\mathsf{m}}(c_1)\pi^{\mathsf{m}}(c_2)$. This defines the average length $L_S$ beyond which correlations in domain ordering can be neglected. $L_S$ is thus the average size of clusters of domains of the same type.

## 1.2 Derivation of symmetries

We start by showing the validity of the extended Chargaff symmetry $P(X) = P(\hat{X})$ for $\ell < L_M$. We denote by $\#_{(\underline{c},\underline{l})}(X)$ the counts of $X$ inside $(\underline{c}, \underline{l})$. Using $\pi[(\underline{c}, \underline{l})] = \pi[\widehat{(\underline{c}, \underline{l})}]$ and $f_C(X) = f_{\hat{C}}(\hat{X})$ we have that $\#_{(\underline{c},\underline{l})}(X) = \#_{\widehat{(\underline{c},\underline{l})}}(\hat{X})$. Finally, for $X$ of size $\ell_X \ll L_M$, the counts of $X$ in the full sequence is dominated by $X$ not overlapping different macro-structures and thus we conclude that ($N' = N - \ell$)

$$
\begin{aligned}
P(X) &\simeq 1/N' \sum_m \sum_{(\underline{c},\underline{l})_m} \#_{(\underline{c},\underline{l})}(X) \\
&= 1/N' \sum_m \sum_{\widehat{(\underline{c},\underline{l})}_m} \#_{\widehat{(\underline{c},\underline{l})}}(\hat{X}) \\
&\simeq P(\hat{X}). \qquad (2)
\end{aligned}
$$

We now show the validity of the nested hierarchy of symmetries discussed in the main paper. We focus on observables of the form $Y = (X_A, X_B; \ell)$, where $X_A$ and $X_B$ are oligonucleotide of size much smaller than typical domain sizes $L_D$. We always approximate the counts of $X$ inside a domain of type $C$ and of length $l$ by $l \cdot f_C(X)$.

Define

$$
\begin{aligned}
\#_{(i)}(Y) &:= \text{number of } Y \text{ fully inside the } i\text{-th domain} \\
\#_{(ij)}(X_A, X_B, \ell) &:= \text{number of } X_A \text{ fully in the } i\text{-th and } X_B \text{ in the } j\text{-th domains, at distance } \ell \\
\#(Y) &:= \text{number of } Y := (X_A, X_B, \ell) \text{ in the full string} \\
&= \sum_i \#_{(i)}(Y) + \sum_i \sum_{j>i} \#_{(ij)}(X_A, X_B, \ell) +
\end{aligned}
$$

$+\{\text{terms where } X_A \text{ or } X_B \text{ overlap domains boundaries}\}$

As we will consider only the case $l_{X_A}, l_{X_B} \ll L_D$, we neglect the last term.

We can now investigate and rule out the main contributions to the overall counting $\#(Y)$ at different scales:

- $(\ell \ll L_D)$: At these scales the following sum dominates,

$$\begin{aligned}
\#(Y) \simeq \sum_{i=1} \#_{(i)}(Y) &\simeq \sum_{m=1}^{r} g_m(\ell)\left[f_{C_\mathbf{m}}(Y) + f_{\hat{C}_\mathbf{m}}(Y)\right] \\
&= \sum_{m=1}^{r} g_m(\ell)\left[f_{C_\mathbf{m}}(Y) + f_{C_\mathbf{m}}(\hat{Y})\right]
\end{aligned}$$

where

$$g_m(\ell) := \frac{1}{2}\sum_{l=\ell}^{\infty} p_m(l)(l-\ell) \qquad\qquad \ell \ll L_D.$$

We conclude that $\#(X_A, X_B, \ell) \simeq \#(\hat{X}_B, \hat{X}_A, \ell)$ at these scales, and thus symmetry $S1$ is valid. This can also be derived directly from equation (2).

For $\ell >> L_D$, $X_A$ and $X_B$ typically lie in different domains and therefore the second term in equation (3) dominates

$$\#(Y) \simeq \sum_{i=1}\sum_{j>i} \#_{(ij)}(X_A, X_B, \ell).$$

The counts will be estimated as the product of the probabilities of $X_A$ and $X_B$ because each domain is an independent realisations. At different scales $\ell$ there are different relationships between the domains in which $X_A$ and $X_B$ typically lie, leading to the following cases:

- $(L_D << \ell < L_S)$: At these scales the sum is dominated by counts of $Y$ inside a cluster of domains of the same type. Each cluster contribute to the counts of $Y$ with a term

$\pi[\{c,l\}](l-\ell)f_C(X_A)f_C(X_B)$ and thus, in this case

$$\begin{aligned}\sum_{i=1}\sum_{j>i}\#_{(ij)}(X_A,X_B,\ell) &\simeq \sum_{m=1}^{r} h_m(\ell)\left[f_{C_\mathbf{m}}(X_A)f_{C_\mathbf{m}}(X_B)+f_{\hat{C}_\mathbf{m}}(X_A)f_{\hat{C}_\mathbf{m}}(X_B)\right]\\ &= \sum_{m=1}^{r} h_m(\ell)\left[f_{C_\mathbf{m}}(X_A)f_{C_\mathbf{m}}(X_B)+f_{C_\mathbf{m}}(\hat{X}_A)f_{C_\mathbf{m}}(\hat{X}_B)\right]\end{aligned}$$

where

$$h_m(\ell):=\frac{1}{2}\sum_{l=\ell}^{\infty}\pi[\{c,l\}](l-\ell) \qquad L_D<\ell\ll L_S$$

We conclude that $\#(X_A,X_B,\ell)\simeq\#(\hat{X}_B,\hat{X}_A,\ell)\simeq\#(X_B,X_A,\ell)\simeq\#(\hat{X}_A,\hat{X}_B,\ell)$ at these scales, and thus symmetry $S2$ (and $S1$) is valid. If the processes are such that correlations inside domains vanishes at a scale smaller than the realization of the process, we consider this shorter correlation time to be the effective domain size $L_D$ and $S2$ sets in at this shorter scale.

- ($L_S\ll\ell\ll L_M$): At these scales the sum is dominated by $X_A$ and $X_B$ lying in different cluster

$$\begin{aligned}\sum_{i}\sum_{j>i}\#_{(ij)}(X_A,X_B,\ell) &= s(\ell)\sum_{m=1}^{r}\Big[f_{C_\mathbf{m}}(X_A)f_{C_\mathbf{m}}(X_B)+f_{C_\mathbf{m}}(X_A)f_{\hat{C}_\mathbf{m}}(X_B)+\\ &\qquad +f_{\hat{C}_\mathbf{m}}(X_A)f_{C_\mathbf{m}}(X_B)+f_{\hat{C}_\mathbf{m}}(X_A)f_{\hat{C}_\mathbf{m}}(X_B)\Big]\\ &= s(\ell)\sum_{m=1}^{r}\Big[f_{C_\mathbf{m}}(X_A)f_{C_\mathbf{m}}(X_B)+f_{C_\mathbf{m}}(X_A)f_{C_\mathbf{m}}(\hat{X}_B)+\\ &\qquad +f_{C_\mathbf{m}}(\hat{X}_A)f_{C_\mathbf{m}}(X_B)+f_{C_\mathbf{m}}(\hat{X}_A)f_{C_\mathbf{m}}(\hat{X}_B)\Big]\\ &= s(\ell)\sum_{m=1}^{r}\left[\left(f_{C_\mathbf{m}}(X_A)+f_{C_\mathbf{m}}(\hat{X}_A)\right)\left(f_{C_\mathbf{m}}(X_B)+f_{C_\mathbf{m}}(\hat{X}_B)\right)\right]\end{aligned} \tag{3}$$

where

$$s(\ell) \simeq \frac{1}{4}(L_M - \ell) \qquad\qquad L_S < \ell \ll L_M.$$

We conclude that $\#(X_A, X_B, \ell) \simeq \#(\hat{X}_B, \hat{X}_A, \ell) \simeq \#(X_B, X_A, \ell) \simeq \#(\hat{X}_A, \hat{X}_B, \ell) \simeq \#(\hat{X}_A, X_B, \ell) \simeq \#(\hat{X}_B, X_A, \ell) \simeq \#(X_B, \hat{X}_A, \ell) \simeq \#(X_A, \hat{X}_B, \ell)$ at these scales, and thus symmetry $S3$ (and $S2, S1$ and $S4$) is valid.

- ($L_M \ll \ell$): At these scales the sum is dominated by counts where $X_A$ and $X_B$ are in different macro-structures:

$$\begin{aligned}\sum_i \sum_{j>i} \#_{(ij)}(X_A, X_B, \ell) &= \sum_{m=1}^{r} \sum_{n>m} q_{m,n}(\ell) \left[ \left( f_{C_{\mathbf{m}}}(X_A) + f_{\hat{C}_{\mathbf{m}}}(X_A) \right) \left( f_{C_{\mathbf{n}}}(X_B) + f_{\hat{C}_{\mathbf{n}}}(X_B) \right) \right] \\ &= \sum_{m=1}^{r} \sum_{n>m} q_{m,n}(\ell) \left[ \left( f_{C_{\mathbf{m}}}(X_A) + f_{C_{\mathbf{m}}}(\hat{X}_A) \right) \left( f_{C_{\mathbf{n}}}(X_B) + f_{C_{\mathbf{n}}}(\hat{X}_B) \right) \right].\end{aligned}$$

where $q_{m,n}(\ell)$ counts how many sites separated by $\ell$ lie in macro-structures m and n, respectively.

We conclude that $\#(X_A, X_B, \ell) \simeq \#(\hat{X}_A, X_B, \ell) \simeq \#(X_A, \hat{X}_B, \ell) \simeq \#(\hat{X}_A, \hat{X}_B, \ell)$ and thus symmetry $S4$ is valid.
